# A Novel Knee Traction Technique to Treat Chronic Knee Pain

**DOI:** 10.7759/cureus.43526

**Published:** 2023-08-15

**Authors:** Leonid Tafler, Samira Ovshaev, Siam Ayon, Amanda Luu

**Affiliations:** 1 Primary Care, Touro College of Osteopathic Medicine, New York, USA; 2 Family Medicine, New York Institute of Technology (NYIT), New York, USA; 3 Osteopathic Manipulative Medicine, Touro College of Osteopathic Medicine, New York, USA

**Keywords:** degenerative knee, manipulation under anesthesia, novel treatment, osteopathic technique, persistent knee pain

## Abstract

Chronic knee pain is one of the most common complaints in primary care offices. Osteopathic family physicians Leonid Tafler and Samira Ovshaev have worked tirelessly for over 10 years to develop a revolutionary and unique technique for treating chronic knee pain. This technique is easy to learn and can be performed at any outpatient practice. This unique technique involves knee traction, which can be performed under anesthesia with one or two consecutive days of treatment, or it can be performed with the assistance of a supportive device in more than two therapy sessions without anesthesia. This new technique has the potential to become a first-line noninvasive technique for chronic knee pain that can go a long way in reducing the burden of debilitating knee pain for patients and their loved ones. This case report aims to describe a few cases of chronic knee pain successfully treated in the office-based surgical center by osteopathic physicians using this innovative technique. Throughout the last 10 years, patients have reported remarkable improvement in their knee pain following treatment with this technique, which has, in turn, significantly increased their quality of life.

## Introduction

Chronic knee pain can be severely debilitating, significantly reducing a person’s ability to perform activities of daily living (ADL) and decreasing their quality of life. Chronic knee pain can affect up to 25% of adults [1] and can occur following a traumatic event or an atraumatic event [1,2]. Some atraumatic causes of chronic knee pain include osteoarthritis (OA), runner’s knee, and arthrofibrosis [3-5]. One of the causes of chronic knee pain can be the development of scar tissue, which can happen over time due to inflammation [5]. 

Current literature emphasizes a stepwise method of treatment to manage chronic knee pain, which includes starting with conservative methods such as nonsteroidal anti-inflammatory drugs (NSAIDs), rest, ice, wearing knee support, physical therapy, and osteopathic manipulation [6]. In some severe cases, surgery can also be considered to treat chronic knee pain [6].

Our article presents three cases of chronic knee pain treated with the unique knee traction technique successfully developed by highly skilled osteopathic physicians. The knee traction technique can be applied with or without anesthesia. The first case is a female who had developed chronic knee pain after a skiing injury at age 28 and came to our osteopathic primary care with chronic knee pain 15 years later. The second case is a 75-year-old male who presented to our osteopathic primary care setting to manage his severe osteoarthritis. The final case is a 61-year-old male who had chronic patellar tendinosis and came to our clinic about five months after the pain started. All three patients were very successfully treated using the novel knee traction technique.

## Case presentation

Case one 

A female with no significant past medical history presented with chronic knee pain due to a skiing injury at the age of 28. In 1994, the patient sustained a severe injury to the left knee while skiing, which resulted in a medial meniscus tear. Immediately after the accident, the patient was able to walk with a limp, but due to her occupation, she had to remain on her feet all day and was unable to rest. She dealt with this debilitating pain for 15 years, which greatly impacted her ADLs such as running and using the stairs. 

The patient tried various forms of physical therapy in hopes of relieving pain and restoring the function of her left knee. She tried NSAID medications, multiple intra-joint injections, and wrapping with mild improvement. However, none of these traditional therapies yielded significant results. She was able to accommodate the discomfort but would be reminded of the injury when certain foot positions would cause her such debilitating pain that she would fall to the ground, which ultimately led her to seek further care. Fifteen years later, in 2009, she was evaluated in our osteopathic office and was suspected of having multiple scar tissue developments in her knee joint because of the chronic inflammation she had been living with. After a thorough evaluation, she was offered manipulation under anesthesia (MUA) and agreed to undergo the procedure. The physicians performed their novel knee traction technique under anesthesia, and the results were incredibly promising. 

The patient reported significant improvement immediately after the procedure and was enthralled to be back to normal in her pre-injury state. She was able to participate in aerobic activities at the gym and go about her life pain-free. The procedure achieved a full range of motion of the left knee after breaking through the scar tissue. The patient reported that she was in this functional state for more than 10 years after the procedure. 

Case two

A 75-year-old male with no significant past medical history presents with chronic left knee pain secondary to severe osteoarthritis with no knee deformity. The patient was seeking a second opinion for his severe medial meniscus arthritis, which was recommended for a left knee replacement by an orthopedic surgeon. The patient’s ADLs were impacted by his knee condition; he could walk on planar surfaces without difficulty, but using the stairs of his apartment proved to be a great challenge. The pain was debilitating enough to provoke the patient to seek care. 

Initially, conservative approaches such as traditional osteopathic therapy and physical therapy in the form of ultrasound and electrical stimulation, exercise, intra-joint steroid injection, and platelet-rich plasma (PRP) injection were applied with little to no benefit. The patient also mentioned that previous therapy at a prior practice included injection with hyaluronic acid before he came to us. After careful consideration of the risks and benefits of the surgery, he decided to explore other conservative options, which led him to our osteopathic family practice to discuss MUA. 

The patient underwent MUA with remarkable improvement. The patient was able to use his staircase without any discomfort, and his left knee range of motion was greatly improved. While the procedure does not relieve arthritis, it improved the range of motion and the patient’s ADLs to the point that a pain-free interval was achieved. Moving forward, we suggest multiple procedures under MUA to build upon this improvement and maintain symptomatic control. As an alternative therapy for severe osteoarthritis, the aim was to improve the overall quality of life. In addition, previous patients have also shown significant improvement with the delayed need for surgical intervention. 

Case three

A 61-year-old male with no significant medical history presented with chronic right knee pain that started about five months ago after prolonged exposure to cold wind. Due to the stiffness in his knees, he tried to stretch by doing a couple of laps in the swimming pool. However, he achieved no relief, and the knee became even more stiff and painful. From then onwards, the pain progressively worsened and limited his mobility, with significant pain going up and down stairs.

After the initial pain began, the patient underwent standard therapy with no improvement for five months. X-ray performed outpatient and musculoskeletal (MSK) ultrasound performed at our office were unremarkable. Our clinic diagnosed him with chronic tendinosis and decided to help break up the scar tissue around his knee to provide comfort. The patient was offered the novel knee traction technique using the traction device without anesthesia. The patient felt immediate relief after 20 minutes of the traction device stretch. The patient could go up and down the stairs with significantly less discomfort after the procedure.

The patient followed up with our clinic twice and received the same knee traction technique without anesthesia, leading to a remarkable resolution of symptoms. After two sessions, the patient could ambulate up and down stairs without any difficulty and could once again drive a car for a long distance. This noninvasive traction technique allowed him to return to his previous level of functioning.

All three patients described above had a history of standard therapy that included a combination of physical therapy, intra-articular injections, NSAIDs, and ice, with minimal improvement. In most cases of chronic knee pain, even those considered surgical candidates are usually referred for multiple physical therapy sessions. However, multiple sessions of physical therapy can be time-consuming. The novel knee traction technique introduced in this article is usually done once or twice under anesthesia, which is a lot less time-consuming and can lead to the remarkable resolution of symptoms.

## Discussion

Technique description 

**Figure 1:**
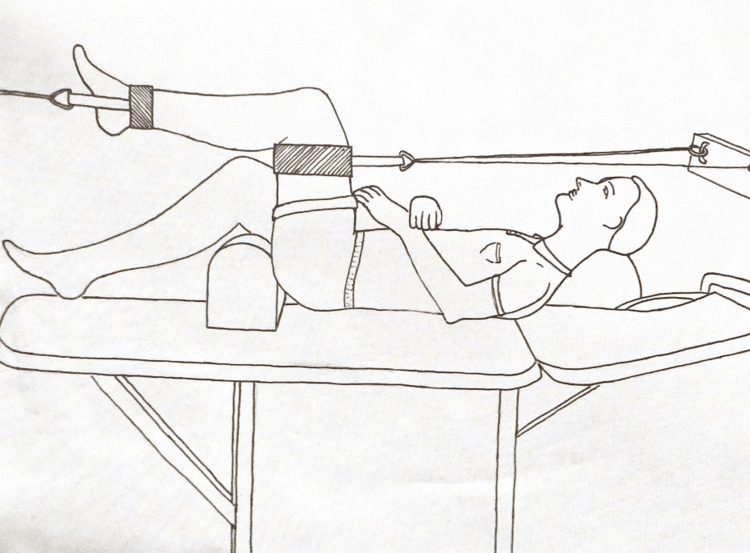
Knee traction device.

Osteopathic knee traction technique under anesthesia: Firstly, the patient lies supine. After introducing propofol narcosis, the affected knee is placed at a 90-degree angle. The physician monitors the patient’s patella. Another physician or healthcare professional stands towards the head of the table and holds onto the manual traction device. A third healthcare provider or physician stands towards the foot and holds it. Maintaining the knee at a femoral, tibial angle of 90 degrees (Figure 1), the provider standing at the top of the table and the provider standing at the bottom of the table pull the affected limb in the opposite direction. This isometric stretch is usually held for 10-15 seconds, and the physician monitoring the patella feels scar tissue release as they feel a type of “crackling movement” under their hand. The treatment is completed once the physician feels the release of scar tissue. Once the patient wakes up from anesthesia, the physician reassesses them. If the physician deems it necessary, a repeat procedure can be performed.

Osteopathic knee traction technique without anesthesia: The patient also lies supine for this technique. Using a traction device, the affected knee is maintained at a femoral, tibial angle of 90 degrees (Figure 1) for 15 minutes with 20 pounds of weight. If needed, this procedure can be repeated once or twice weekly for up to three to 10 sessions. 

While osteopathic manipulative medicine (OMM) is traditionally performed when the patient is awake, MUA is a treatment modality that more physicians have started to utilize. OMM under anesthesia is very effective for the treatment of chronic conditions. MUA facilitates muscle relaxation, helps with patient comfort during manipulation, and helps break up scar tissue from chronic inflammation [7,8]. MUA allows the physician to stretch and manipulate muscle tissue and joints without the patient’s involuntary resistance to motion. When the patient is sedated with propofol, they can still feel pain in the affected joint. Due to the relaxation of the muscles and the patient’s pain, which can be visualized by facial expression, physicians can determine the extent of the range of motion during the procedure. This allows for a very safe and effective procedure [9].

Chronic knee pain continues to affect a large percentage of the adult population. Osteoarthritis is expected to become the fourth-leading cause of disability in the coming decades [10]. Several types of surgery are often considered a treatment modality for chronic knee pain. Based on the available evidence, arthroscopic lavage provides only short-term benefits to selected patients with mild OA. The literature recommends against arthroscopic debridement as a routine treatment for chronic knee pain [10]. Osteotomies efficiently reduce pain and improve function for younger patients and have been accepted as a safe treatment modality. However, no studies have compared it with placebo or conservative treatment alone [10]. Total knee arthroplasty is considered adequate for end-stage arthritis of the knee. However, postoperative pain persists in one out of eight patients [11]. In addition, based on two high-quality randomized controlled trials, arthroscopic surgery offered mild benefits for most patients with knee osteoarthritis [12].

## Conclusions

Our case report introduces an osteopathic technique performed under anesthesia that can benefit many patients suffering from knee pain due to scar tissue formation from chronic inflammation. In this article, we discussed three cases; one case of a ski accident that resulted in over 15 years of chronic inflammation, one case of severe osteoarthritis, and the last case was a patient who had chronic patellar tendinosis. The novel osteopathic therapeutic technique under anesthesia was applied successfully to the first two cases, and without anesthesia, it was applied to the third patient with significant improvement. This newly developed procedure is important because, in the case of chronic inflammation, scar tissue develops, and in order to break down this scar tissue to restore joint mobility, osteopathic manipulation under anesthesia has been proven to be successful. However, it is important to consider that even though propofol is rapidly cleared from circulation, it can still lead to respiratory depression, blurry vision, and heart rate changes, among other things. Patients need to be monitored carefully while under narcosis and after they are awakened. In one or two sessions under anesthesia with a supportive device, an immediate effect is seen, which reduces therapy time as opposed to multiple sessions of physical therapy. This efficient technique can also be used in combination with other therapies or alone in order to achieve rapid relief in minimal time. Compared to current modalities of treatment, such as arthroscopic debridement, osteotomies, and total knee arthroscopy, our approach allows for a less invasive procedure that can immediately alleviate symptoms and provide patient comfort. In the stepwise approach of chronic knee pain treatment, using this technique can be a great alternative before more invasive treatments, such as surgery, are considered. This technique is widely applicable to different etiologies of chronic knee pain, as shown by our cases, and can be performed by any trained healthcare professional.
